# Losartan treatment attenuates hindlimb unloading-induced atrophy in the soleus muscle of female rats via canonical TGF-β signaling

**DOI:** 10.1186/s12576-022-00830-8

**Published:** 2022-03-09

**Authors:** Toshinori Yoshihara, Mizuki Takaragawa, Shohei Dobashi, Hisashi Naito

**Affiliations:** 1 Graduate School of Health and Sports Science, Juntendo University, Chiba, Japan; 2 Institute of Health and Sports Science & Medicine, Juntendo University, Chiba, Japan

**Keywords:** Sex difference, Muscular atrophy, Angiotensin receptor blocker, TGF-β signaling, Hindlimb unweighting

## Abstract

We investigated the protective effect of losartan, an angiotensin II type 1 receptor blocker, on soleus muscle atrophy. Age-matched male and female Wistar rats were subjected to hindlimb unloading, and the soleus muscle was removed on days 1 and 7 for analysis. Females showed greater reductions in relative weight and myofiber cross-sectional area of the soleus muscle than males on day 7 post-hindlimb unloading. Losartan partially protected females against muscle atrophy. Activation of the canonical TGF-β signaling pathway, assessed via Smad2/3 phosphorylation, was lower in females following losartan treatment and associated with lower levels of protein ubiquitination after 1 (myofibril) and 7 (cytosol) days of unloading. However, no effect was observed in non-canonical TGF-β signaling (p44/p42 and p38 MAPK phosphorylation) in males or females during unloading. Our results suggest that losartan provides partial protection against hindlimb unloading-induced soleus muscle atrophy in female rats, possibly associated with decreased canonical TGF-β signaling.

**Supplementary Information:**

The online version contains supplementary material available at 10.1186/s12576-022-00830-8.

## Background

The physiology and hormonal profiles of males and females differ, which influence muscle adaptation during disuse. For example, a previous study demonstrated that 17 weeks of horizontal bed rest causes a greater reduction in whole muscle volume in women (− 17.3%) than in men (− 10.7%) [1]. Moreover, women show a greater decline in thigh muscle size than men, even after shorter periods of bed rest (− 21% in 60 days in women vs. − 17% in 84 days in men) [2–5]. We have recently reported that 7 days of hindlimb unloading causes a higher reduction in the relative soleus muscle weight and fiber cross-sectional area in female than in male rats, which is associated with different responses to the FoxO3a/ubiquitin–proteasome system via the canonical transforming growth factor-beta (TGF-β) signaling pathway [6]. The canonical TGF-β signaling pathway uses Smad2 and Smad3 to transfer signals. Smad2/3 are directly phosphorylated by transforming growth factor-beta receptor 1 and translocate to the nucleus to regulate gene transcription [7]. Female rats exhibit a slight increase in myostatin expression with greater activation of the downstream Smad2/3 signaling compared with male rats, and the latter show nonsignificant muscle decline following unloading. Thus, suppressing TGF-β activation pathway may be a useful strategy to effectively attenuate disuse-induced muscle atrophy in females.

Losartan, an angiotensin II type 1 (AT1) receptor blocker, inhibits canonical TGF-β signaling activation and has been shown to promote muscle remodeling in mouse models of dystrophin-deficient Duchenne muscular dystrophy [8]. Furthermore, Burks et al. [9] demonstrated that 1 week of losartan treatment, prior to and during immobilization-induced muscle atrophy, prevents the disuse-related muscle atrophy of aged mice by modulating the canonical and non-canonical TGF-β signaling cascades. In addition, recent evidence has shown that losartan attenuates hindlimb unloading-induced soleus muscle atrophy via the partial prevention of reactive oxygen species production, which is linked to the suppression of active Nox2 [10], suggesting that losartan also prevents disuse-induced muscle atrophy.

Losartan treatment reduces disuse-induced muscle atrophy by modulating the canonical and non-canonical TGF-β signaling pathways; hence, it may also protect female rats against disuse-induced soleus muscle atrophy. However, there is no evidence suggesting that losartan attenuates the activation of canonical (Smad2/3) and non-canonical (p44/42 MAPK) TGF-β signaling in atrophied female rat soleus muscle. Therefore, in this study, we aimed to examine the effects of losartan treatment on muscle atrophy, in male and female rats, as well as its sex-specific effects on TGF-β signaling in skeletal muscle atrophy induced by hindlimb unloading.

## Methods

### Experimental animals and hindlimb unloading

Male and female Wistar rats (3–4 weeks old; *n* = 35/sex) were housed in a climate-controlled room (23 °C ± 1 °C, 55% ± 5% relative humidity, and 12:12 h light/dark cycle) with free access to standard diet and water, during the entire duration of the study. After acclimation (at 10 weeks of age), both male (251.6 ± 7.1 g) and female (150.1 ± 5.7 g) rats were divided into age-matched groups: control group not subjected to hindlimb unloading (HU)(PRE; *n* = 7/group), HU-control group (CON; *n* = 14/group), and HU losartan administration (LOS) group (*n* = 14/group). The CON and LOS groups were subjected to hindlimb unloading on day 1 or 7, as previously described [11]. The rats were anesthetized with pentobarbital sodium (60 mg/kg) and isoflurane (~ 3%–5%), and euthanized by pentobarbital sodium overdose (150 mg/kg) and cardiac resection. The soleus muscle was carefully removed before hindlimb (PRE) and on day 1 (*n* = 7/group) or 7 (*n* = 7/group) after hindlimb unloading, and weighed. The muscles were flash-frozen in liquid nitrogen and stored at − 80 °C until analysis.

All protocols were approved by the Juntendo University Animal Care Committee (2019–16, approved on April 13th, 2020) and followed the principles for the care and use of laboratory animals set by the Physiological Society of Japan.

### Losartan administration

The LOS groups (male and female) received an intraperitoneal priming dose of losartan (20 mg/kg), and the drug was also added to the water and provided ad libitum (0.3 g/L; Losartan Potassium, Tokyo Chemical Industry UK Ltd., Tokyo, Japan) during unloading (one rat per cage); the losartan treatment continued until the end of the experiment. The placebo control group (CON) received an intraperitoneal injection of aseptic saline and were allowed to drink water without losartan ad libitum. The total water intake of each rat during unloading was monitored and total losartan intake and dosage per rat were calculated (Additional file 2: Table S1).

### Myofiber cross-sectional area

To assess the soleus muscle fiber myofiber cross-sectional area (CSA), the frozen soleus muscle (flash-frozen at resting length) was sliced into 10-μm segments (CM3050S, Leica, Wetzlar, Germany) and stained for dystrophin visualization (#RB-9024-R7; Thermo Scientific, Waltham, MA, USA). The ImageJ software package (1.53 k) was used to determine fiber CSA by measuring the CSA of 300–600 fibers (National Institutes of Health, Bethesda, MD, USA) in each muscle sample.

### Muscle preparation

Frozen soleus muscle tissue was powdered and a 20–30 mg sample was homogenized in ice-cold homogenization buffer (20 mM HEPES pH 7.4, 4 mM EGTA, 0.1 mM ethylenediaminetetraacetic acid (EDTA), 10 mM MgCl_2_, and 1% Triton X-100) containing cOmplete EDTA-free protease (Roche, Penzberg, Germany) and PhosSTOP phosphatase (Roche) inhibitor cocktails, as previously described [12]. The lysate was then centrifuged at 12,000×*g* for 15 min at 4 °C, and the supernatant was collected.

To obtain the myofibril fraction [6], the insoluble particulate fraction was washed several times in ice-cold homogenization buffer and centrifuged at 12,000×*g* for 5 min at 4 °C. The pellet was resuspended in lysis buffer (20 mM HEPES pH 7.4, 150 mM NaCl, 1% (w/v) lithium dodecyl sulfate, 1% (v/v) Nonidet P-40) and centrifuged at 17,000×*g* for 5 min at 4 °C. The concentration of the fractionated proteins was measured using a bicinchoninic acid protein assay kit (Thermo Fisher Scientific).

### Immunodetection

Equal amounts of protein (5–10 μg) were loaded onto a precast 4%–15% Tris–glycine extended polyacrylamide gel (Bio-Rad, Copenhagen, Denmark) and the separated proteins were transferred to a polyvinylidene difluoride membrane (Bio-Rad) that was incubated in blocking buffer (EveryBlot; Bio-Rad) for 20 min. After several washes, the membrane was incubated with the following primary antibodies. The primary antibodies against phosphorylated Ser465/467-Smad2/Ser423/425-Smad3 (catalog #8828, 1:2,000), Smad2/Smad3 (#8685, 1:2,000), phosphorylated Thr202/Tyr204-p44/42 MAPK (Erk1/2) (#4370, 1:2,000), ERK (#4695, 1:2,000), phosphorylated Thr180/Tyr182 p38 MAPK (#4511, 1:2000), and p38 MAPK (#8690, 1:2000) were purchased in Cell Signaling Technology (Beverly, MA, USA); the anti-mono- and poly-ubiquitinated conjugates (BML-PW8810, 1:2,000) and superoxide dismutase 1 (SOD-101, 1:10,000) antibodies were obtained from Enzo Therapeutics (Farmingdale, NY, USA); anti-growth differentiation factor (GDF)-8/myostatin (251651, 1:2000) was purchased from Abbiotec (San Diego, CA, USA); anti-ACVR2B (PB9975, 1:2000) was obtained from BOSTER Bio (Pleasanton, CA, USA); TGFβR2 (sc-17799, 1:2000) was purchased from Santa Cruz (Dallas, TX, USA); and anti-AGTR1 (GTX63229, 1:2000) was obtained from GeneTex (San Diego, CA, USA). Subsequently, the membranes were incubated with anti-rabbit or anti-mouse horseradish peroxidase-conjugated secondary antibodies (1:10,000 or 1:5000), from Cell Signaling Technology (Beverly, MA, USA) in dilution buffer (3% bovine serum albumin (BSA)/Tris-buffered saline with Tween 20) for 1 h at 25–26 °C. After several washes, the protein bands were visualized using the enhanced chemiluminescence Prime reagent (Amersham, Piscataway, NJ, USA), and the signal was recorded using a ChemiDoc Touch imaging system (Bio-Rad). Signal intensity was analyzed using Image Lab v.5.2.1 software (Bio-Rad). Revert 700 Total Protein Stain (LI-COR Biosciences, Lincoln, NE, USA) was used to normalize protein expression.

To evaluate protein oxidation, we used OxiSelect Protein Carbonyl Immunoblot Kit (STA-308; Cell Biolabs, Inc. Huissen, Netherlands) according to the manufacturer’s recommended procedure. The signal was captured using the ChemiDoc Touch imaging system (Bio-Rad) and Revert 700 Total Protein Stain (LI-COR Biosciences) was used as a loading control.

### Real-time polymerase chain reaction

Total RNA isolation and cDNA synthesis were performed as previously described [6]. mRNA levels for both atrogin-1/MAFbx (Rn00591730_m1) and MuRF1 (Rn00590197_m1) were determined using a TaqMan gene expression assay (Applied Biosystems, Foster City, CA, USA) and normalized to 18S mRNA expression. Relative changes (ΔΔCt) in the expression levels of atrogin-1 and MuRF1 were determined by subtracting the ΔCt of the male control rat (PRE).

### Statistical analysis

Values are expressed as mean ± standard deviation. The statistical significance of normal-distributed data was evaluated by two-way (sex × group) analysis of variance (ANOVA), at each endpoint (days 1 and 7), and when the interaction was significant simple effects tests were performed. When significant main effects were found (without significant interaction), pairwise comparisons were performed where necessary, using Sidak’s method. For variables that did not show normal distribution after Bartlett’s test, the Kruskal–Wallis test was performed and followed by the two-stage step-up method of Benjamini, Krieger, and Yekutieli; the test was used when significant main effects were found. Unpaired Student’s *t*-tests were used to compare variables between the CON and LOS groups within each sex; *P* < 0.05 was considered statistically significant. All analyses were performed using Prism v.8.0 software (GraphPad Inc., La Jolla, CA, USA).

## Results

### Body weight, soleus muscle weight, and myofiber CSA

**Fig. 1:**
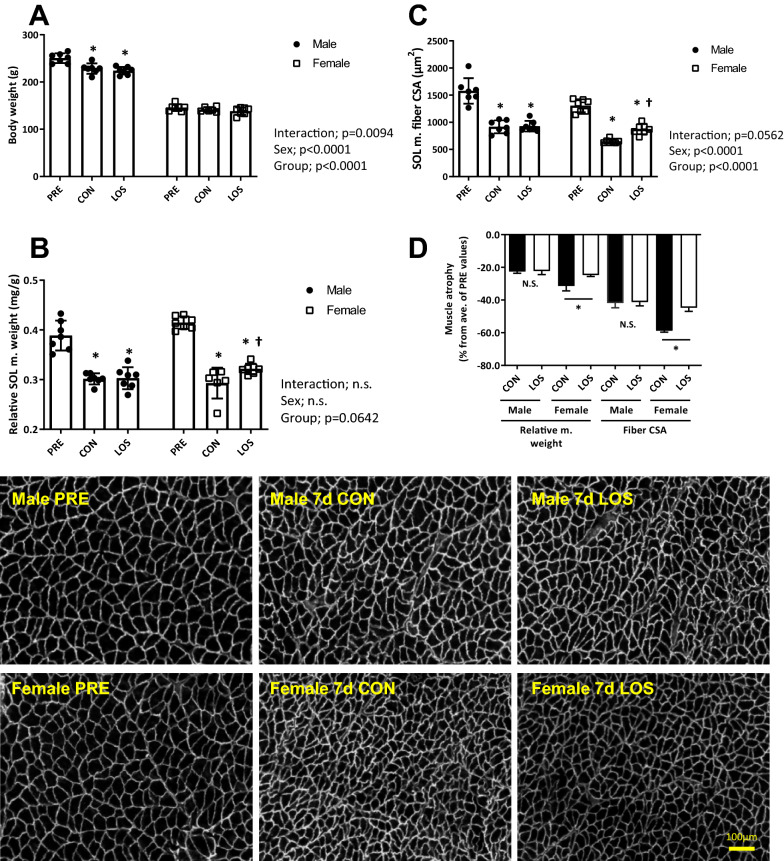
Body weight and soleus muscle phenotype. Body weight (**a**), soleus muscle weight relative to body weight (**b**), muscle fiber cross-sectional area (**c**), and dystrophin staining of rat soleus muscle sections (scale bar = 100 μm) and percent change of atrophy relative to untreated controls (**d**) after 7 days of hindlimb unloading. CON, control; LOS, losartan. Samples were collected 7 days after hindlimb unloading. Values are presented as mean ± standard deviation; *n* = 7 per time point. The data were analyzed using two-way ANOVA. *P < 0.05 vs. PRE and ^†^*P* < 0.05 vs. CON of each sex

Figure 1 shows total body weight and relative soleus muscle weight and myofiber CSA after 7 days of hindlimb unloading. Female rats exhibited a lower body weight loss (Fig. 1a) in response to hindlimb unloading than male rats. However, relative soleus muscle weight and CSA values were significantly reduced in both male (0.389 vs. 0.302 mg/g and 1,578.5 vs. 917.8 μm^2^, *P* < 0.05) and female (0.415 vs. 0.293 mg/g and 1304.6 vs. 651.3 μm^2^, *P* < 0.05) rats on day 7 post-hindlimb unloading (Fig. 1b and c). In addition, the female LOS group showed significantly higher relative soleus muscle weight and CSA values that the female CON group (0.322 vs. 0.293 mg/g, + 10% and 873.3 vs. 651.3 μm^2^, + 13%, *P* < 0.05). Importantly, on day 7 of unloading, CON and LOS male rats showed no differences in relative soleus muscle weight and CSA nor in muscle atrophy (Fig. 1b–d), whereas females showed partial protection from muscle atrophy following losartan treatment based on their relative muscle weight (CON, − 31.4% vs. LOS, − 24.7%; *P* < 0.05) and CSA (CON, − 58.7% vs. LOS, − 44.7%; *P* < 0.05) (Fig. 1d).

### Ubiquitinated protein levels and E3 ligases mRNA levels

**Fig. 2:**
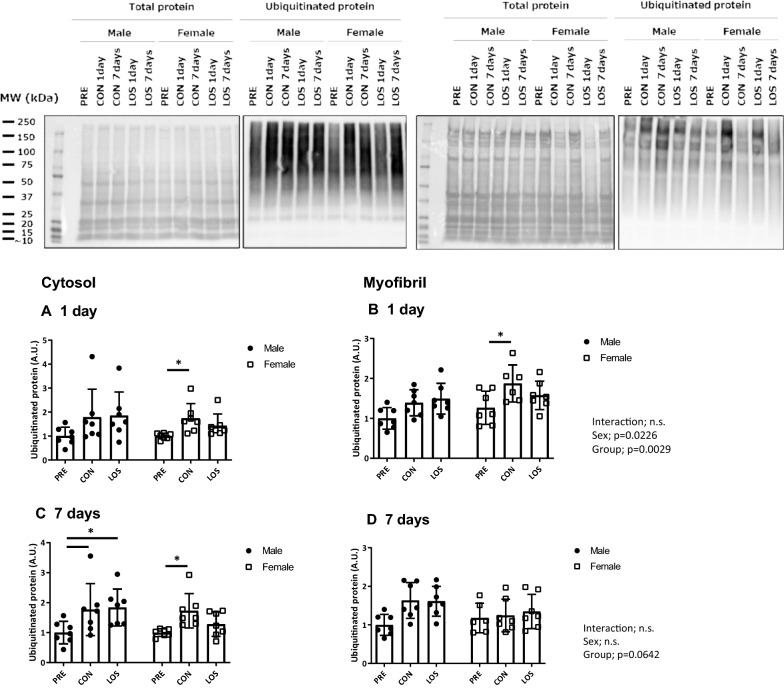
Total and ubiquitinated protein expression on days 1 and 7 following hindlimb unloading. CON, control; LOS, losartan. Ubiquitinated protein expression in the cytosol (**a** and **b**) and myofibril (c and d) before (PRE), and on days 1 and 7 following hindlimb unloading, and representative blots. Values are represented as mean ± standard deviation; *n* = 7 per time point. Normally distributed data were analyzed using two-way ANOVA. For non-normal distribution, the Kruskal–Wallis test was performed, followed by the two-stage step-up method of Benjamini, Krieger, and Yekutieli; the test was used for the analysis within each sex. *P < 0.05 vs. PRE of each sex

Figure 2 shows the ubiquitinated protein levels in the cytosolic and myofibrillar fractions of the soleus muscle on day 1 (Fig. 2a and b) and day 7 (Fig. 2c and d) following hindlimb unloading. In the cytosolic fraction, 7 days of unloading induced a significant increase in ubiquitinated protein expression in male rats in both CON and LOS groups. In contrast, female rats showed a significant increase in ubiquitinated protein expression in the cytosolic fraction only in the CON group after 1 and 7 days of unloading. Regarding the myofibrillar fraction, ubiquitinated protein expression was higher in females than in males 1 day after unloading (Fig. 2c), and significantly higher in CON than in PRE female rats (*P* < 0.05). Moreover, no significant changes were observed in the ubiquitinated protein levels of the myofibrillar fraction on day 7 in both male and female rats (Fig. 2d).

**Fig. 3:**
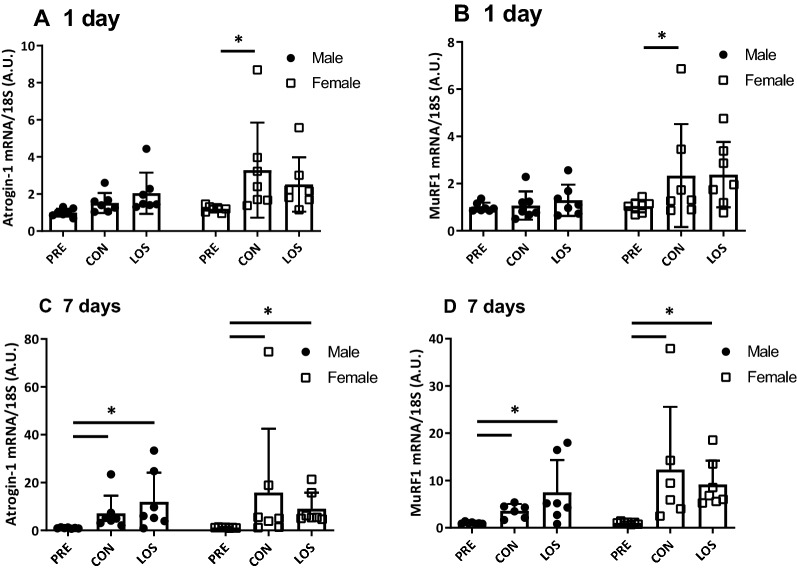
E3 ubiquitin ligase mRNA expression 1 and 7 days after hindlimb unloading. Atrogin-1 (**a**) and MuRF1 (**b**) mRNA expression. CON, control; LOS, losartan. Samples were collected before (PRE), and on days 1 and 7 following hindlimb unloading. Values are expressed as mean ± standard deviation; *n* = 7 per time point. Normally distributed data were analyzed using two-way ANOVA. *P < 0.05 vs. PRE of each sex

Atrogin-1/MAFbx and MuRF1 mRNA level analysis (Fig. 3) showed that the mRNA levels of both genes were significantly increased in CON females on day 1 following unloading (Fig. 3a and b), but not in the LOS group. After 7 days of unloading, both atrogin-1/MAFbx and MuRF1 mRNA levels were significantly increased in both males and females (Fig. 3c and d).

### Changes in canonical TGF-β signaling

**Fig. 4:**
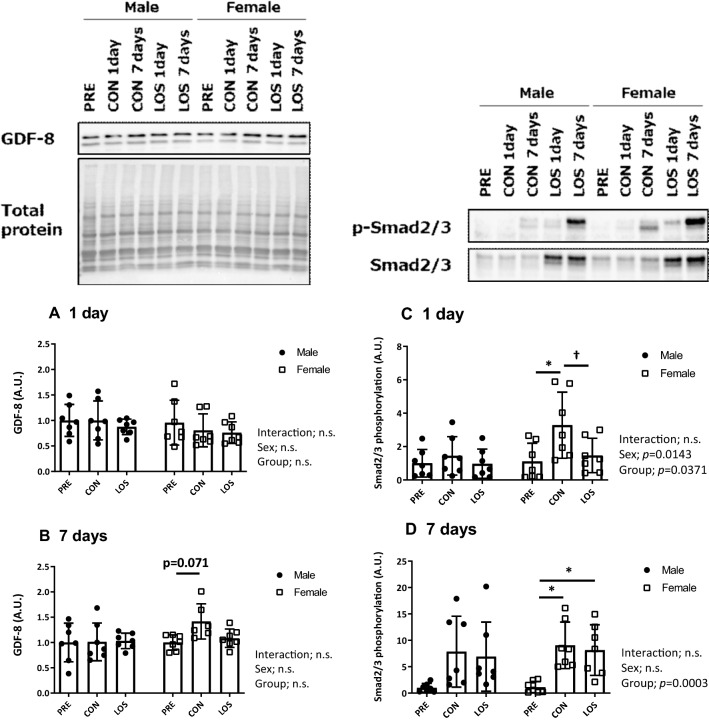
Canonical TGF-β signaling transducer expression. Protein expression of GDF-8/myostatin (**a** and **b**) Smad2 (Ser465/467)/Smad3 (Ser423/425) phosphorylation ratio (c and d) on days 1 and 7 after unloading, and representative blots. CON, control; LOS, losartan. Samples were collected before (PRE), and on days 1 and 7 following hindlimb unloading. Values are expressed as mean ± standard deviation; *n* = 7 per time point. Normally distributed data were analyzed using two-way ANOVA. *P < 0.05 vs. PRE and ^†^*P* < 0.05 vs. CON of each sex

To evaluate changes in the canonical TGF-β signaling pathway, we analyzed GDF-8/myostatin protein levels (Fig. 4a and b), and the phosphorylation status of the mothers against decapentaplegic homolog proteins Smad2 (Ser465/467) and Smad3 (Ser423/425) (Fig. 4c and d), in the soleus muscle during hindlimb unloading. Although GDF-8 protein levels tended to increase in CON females (*P* = 0.071), there were no significant differences between days 1 and 7 post-unloading, in neither male nor female groups.

One day after unloading, there were significant differences in the Smad2 (Ser465/467) and Smad3 (Ser423/425) phosphorylation status between the sexes (*P* = 0.0143) and the groups (*P* = 0.0371) (Fig. 4c). In female rats, the Smad2 (Ser465/467) and Smad3 (Ser423/425) phosphorylation ratio was significantly higher in the CON group than in the PRE group (*P* < 0.05); however, the phosphorylation ratio was significantly reduced by losartan treatment (LOS group; *P* < 0.05). Furthermore, significant differences between the groups (*P* = 0.0003) were observed, and the Smad2 (Ser465/467) and Smad3 (Ser423/425) phosphorylation ratio was significantly higher in the female CON and LOS group than in the PRE group on day 7 after unloading (Fig. 4d).

### Changes in non-canonical TGF-β signaling

**Fig. 5:**
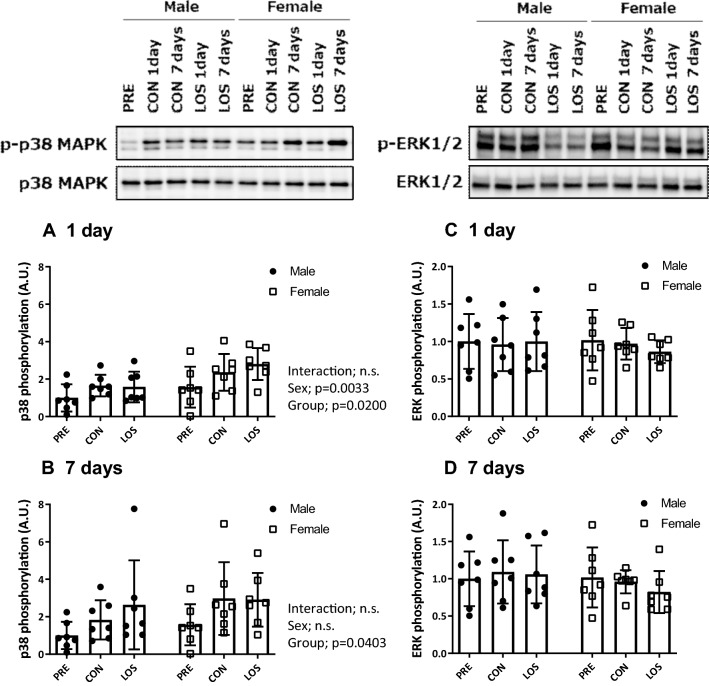
Non-canonical TGF-β signaling transducer expression. Phosphorylation ratio of p38 MAPK (Thr180/Tyr182) (**a** and **b**) and p44/42 MAPK (Erk1/2) (Thr202/Tyr204) (**c** and **d**), and representative blots. CON, control; LOS, losartan. Samples were collected before (PRE), and on days 1 and 7 following hindlimb unloading. Values are expressed as mean ± standard deviation; *n* = 7 per time point. Normally distributed data were analyzed using two-way ANOVA. For non-normal distribution, the Kruskal–Wallis test was performed, followed by the two-stage step-up method of Benjamini, Krieger, and Yekutieli; the test was used for the analysis within each sex

To better assess the changes in the non-canonical TGF-β signaling pathway, we examined the phosphorylation ratio of p38 MAPK (Fig. 5a and b) and ERK (Fig. 5c and d) in the soleus muscles on days 1 and 7 following hindlimb unloading. Significant differences were observed in the p38 MAPK phosphorylation ratio between the sexes (*P* = 0.0033) and the groups (*P* = 0.0200) on day 1 following unloading. Moreover, 7 days following hindlimb unloading, the p38 MAPK phosphorylation ratio was similar in males and females (Fig. 5b), and no significant changes were observed in ERK phosphorylation levels in either sex during unloading.

### Oxidized protein and SOD1 expression levels

**Fig. 6:**
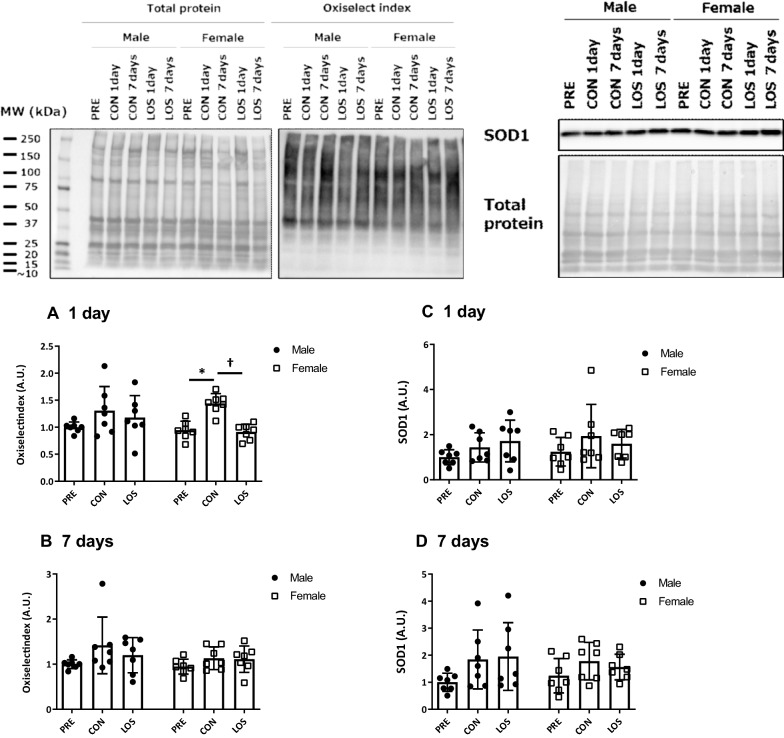
Oxidized protein and superoxide dismutase 1 expression. CON, control; LOS, losartan. Oxidized protein (**a** and **b**) and superoxide dismutase 1 expression (**c** and **d**) on days 1 and 7 after hindlimb unloading. Samples were collected before (PRE), and on days 1 and 7 following hindlimb unloading. Values are represented as mean ± standard deviation; *n* = 7 per time point. Normally distributed data were analyzed using two-way ANOVA. *P < 0.05 vs. PRE of each sex. For non-normal distribution, the Kruskal–Wallis test was performed, followed by the two-stage step-up method of Benjamini, Krieger, and Yekutieli; the test was used for the analysis within each sex

One day after hindlimb unloading, the level of protein carbonyls (i.e., oxiselect index) in the soleus muscle was significantly higher in the CON group than in the PRE group in female rats; however, the increase was suppressed by losartan treatment (Fig. 6a). There was no significant change in protein carbonyls 7 days after unloading (Fig. 6b). No significant changes in SOD1 expression in either sex during unloading were observed (Fig. 6c and d).

### Activin receptor (ACVR) IIB and TGF-β type II receptor (TGFβRII) expression levels

**Fig. 7:**
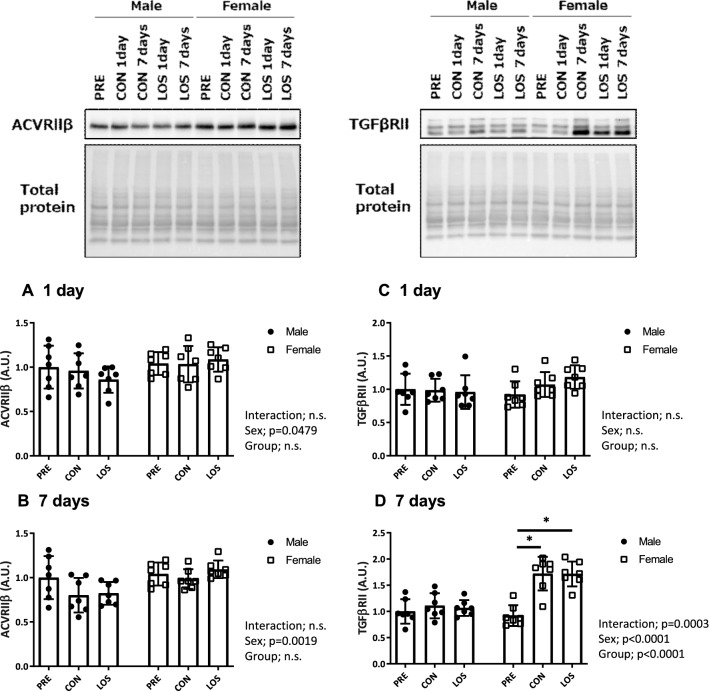
Activin receptor (ACVR) IIB and TGF-β type II receptor (TGFβII) expression levels. Protein expression of ACVR IIB (**a** and **b**) and TGFβII (**c** and **d**) on days 1 and 7 after unloading, and the representative blots. CON, control; LOS, losartan. Samples were collected before (PRE), and on days 1 and 7 after hindlimb unloading. Values are expressed as mean ± standard deviation; *n* = 7 per time point. Normally distributed data were analyzed using two-way ANOVA. *P < 0.05 vs. PRE of each sex

Significant differences were observed in the ACVRIIB level between the sexes on days 1 and 7 after unloading (P = 0.0479 and 0.0019; Fig. 7a and b). Although no significant changes in TGFβRII expression in either sex were observed 1 day after hindlimb unloading (Fig. 7c), TGFβRII expression significantly changed compared with that in the PRE group (P = 0.0003; Fig. 7d). Moreover, TGFβRII expression was significantly higher in the female CON and LOS groups than in the PRE group on day 7 after unloading (Fig. 7d).

### AT1 receptor protein level

No significant changes were observed in the levels of AT1 receptor protein expression in the soleus muscle in either sex during unloading (Additional file 1: Figure S1).

## Discussion

In this study, female rats showed a greater reduction in the relative soleus muscle weight than their male counterparts following hindlimb muscle unloading; however, losartan treatment provided partial protection against this reduction in female rats. Although the specific underlying mechanism behind this remains unclear, our findings suggest that it involves the downregulation of the canonical TGF-β signaling pathway (Smad2/Smad3 phosphorylation) in the soleus muscle of female rats and that losartan inhibits the canonical TGF-β signaling pathway, thereby attenuating muscular atrophy in female rats.

We have previously shown that female rats exhibit greater muscle atrophy associated with muscle disuse than male rats and both show different responses in soleus muscle to the FoxO3a/ubiquitin–proteasome pathway following hindlimb unloading, which may be associated with differences in the GDF-8/myostatin signaling [6]. Thus, the current study examined the effects of losartan on muscle atrophy in female rats and investigated the role of TGF-β signaling in hindlimb unloading-induced skeletal muscle atrophy. The main finding of our study was that female rats exhibited a greater reduction in relative soleus muscle weight and CSA after 7 days of hindlimb unloading; however, losartan treatment attenuated this reduction. Specifically, we found that the activation of canonical TGF-β signaling components (Smad2/Smad3 phosphorylation) was lower in females treated with losartan on days 1 and associated with lower levels of protein ubiquitination on days 1 and 7 following unloading. TGF-β belongs to a family of cytokines that signals through the Smad intracellular signaling cascade, which negatively regulates muscle mass [13, 14]. Moreover, Smad2 and 3 are downstream regulators of GDF-8, which binds to activin type IIB receptor (ActRIIB), thereby inducing Smad2/3 activation (via phosphorylation), and subsequent formation of a complex with Smad4 [15], which in turn translocates into the nucleus and induces gene transcription associated with cell proliferation and differentiation and protein metabolism in muscles [16]. Moreover, the sex differences observed in response to unloading may be attributed to higher levels of *ActRIIB* mRNA in females [17]. Indeed, our data indicated that female rats showed higher levels of ActRIIB protein expression compared with male rats; however, the precise underlying mechanism remains to be determined. Nevertheless, the fact that losartan treatment attenuated the reduction in the relative muscle weight in females suggests that suppressing the AT1 receptor is an effective strategy against disuse-induced skeletal muscle atrophy in female rats by preventing Smad2/3 signaling.

TGF-β can directly induce muscle fiber atrophy and reduce maximum isometric force production in TGF-β-treated mice via atrogin-1 protein upregulation [18], an E3 ubiquitin ligase expressed in skeletal muscle that mediates the polyubiquitination of proteins for proteolysis by 26S proteasome [19]. Muscle protein degradation during disuse is primarily associated with oxidative stress and the downstream ubiquitin–proteasome pathway [20, 21], and the ubiquitination of myofibrillar proteins is a critical step in their degradation [22]. In the present study, we observed that the levels of oxidized and ubiquitinated proteins in the soleus muscle significantly increased in female rats on day 1 after unloading, with significantly higher expression levels of atrogin-1 and MuRF1 mRNA, compared with those in male rats, which were attenuated following losartan treatment, partly owing to the inhibition of canonical TGF-β signaling activation. On the contrary, losartan treatment failed to prevent TGF-β type II receptor (TGFβRII) protein upregulation and regulate the downstream Smad2/3/E3 ligase axis on day 7 after unloading. Thus, TGFβRII-mediated Smad2/3 activation is another approach to protect against disuse muscle atrophy in female rats. Moreover, although the upregulation of TGFβRII during unloading in female rats might support greater muscle atrophy in females than in males, the molecular mechanisms underlying muscle atrophy mediated by TGF-β are not well-understood. Thus, further studies are warranted to determine the precise mechanism underlying losartan-induced inhibition of TGF-β signaling in female rats.

In contrast, losartan treatment had no effect on the activation of non-canonical TGF-β signaling (p44/p42 and p38 MAPK phosphorylation) during unloading. Losartan intake (0.9 g/L in drinking water with a water intake of 3–3.3 mL) does not alter ERK phosphorylation status during limb immobilization in tibialis anterior muscles of aged mice, but significantly reduces p38 MAPK phosphorylation [9]. However, limb immobilization per se did not alter p38 MAPK activation in aged muscle; thus, the effects of losartan treatment during muscle disuse remain unclear.

In addition, no protection against muscular atrophy in male rats during hindlimb unloading was observed in this study, which may be partly attributed to the following reasons. We administered an intraperitoneal priming dose of losartan (20 mg/kg) immediately after starting unloading, and added losartan ad libitum in water (0.3 g/L) during unloading. We assumed that the male and female rats would receive relatively the same amount of losartan during unloading; however, the losartan dosage was 25.9 ± 1.8 mg/kg/day (16.9 ± 1.4 mL/day) for males and 43.1 ± 8.7 mg/kg/day (18.5 ± 4.6 mL/day) for females. Nonetheless, the dosage might not have been associated with the protective effects observed against muscle atrophy (percent change in relative muscle weight and CSA), in both male and female rats (male *r* = − 0.2861 and -0.0689, female *r* = 0.1409 and − 0.216, all *r* = 0.310 and − 0.328, *p* > 0.05). Thus, the influence of the different amounts of losartan consumed during unloading was negligible in the present study. Moreover, sex differences have also been reporting to influence the activation of the FoxO3a/ubiquitin–proteasome pathway following hindlimb unloading [6]; hence, losartan treatment in our study (20–40 mg/kg/day during unloading) would provide partial protection against hindlimb unloading-induced soleus muscle atrophy in females, which may be associated with lowered canonical TGF-β signaling. However, additional studies are required to clarify the sex-specific effects of losartan administered at equal dosages.

Although we demonstrated that losartan treatment provided partial protection against disuse-induced soleus muscle atrophy in female rats, the dosage of losartan in this study was too high for application in humans. The rats were administered 20–40 mg/kg/day losartan during unloading, and it was comparable to the dosage of 1.2–2.4 g/day for a human weighing 60 kg. Generally, an oral administration contains 25–50 mg of losartan potassium (maximum dose of 100 mg once daily), and therefore, we need to re-consider the dosage. We selected 20–40 mg/kg/day losartan potassium administration (0.3 g/L in drinking water) based on previous studies [9, 10], which provided evidence that losartan attenuates hindlimb unloading-induced soleus muscle atrophy via the partial prevention of reactive oxygen species production. Moreover, to alleviate hypertension and lipid metabolism [23] in diabetic rats, and prevent sarcopenia [9], diaphragmatic dysfunction [24], or myopathic state [8], a higher dosage of losartan (25–72 mg/kg/day or 0.6–0.9 g/ L in drinking water) has been used, with no adverse effect on animals. Indeed, there is no data suggesting the optimal dosage of losartan for disuse muscle atrophy, we should carefully consider the adverse effects or the longer-term effects of losartan administration to use it for suppressing muscle atrophy in humans.

In this study, we did not consider the female estrous cycle during unloading, sex hormones (estrogen and testosterone), or other sex differences in hormone concentrations (such as glucocorticoid hormone) that might have affect muscle protein turnover, which is a major limitation. However, as the rat estrous cycle is relatively short, at approximately 4.5 days [25], we assume that every female rat had 1 or 2 estrous cycles (ovulation) during the 7 days of hindlimb unloading. Moreover, it has been reported that hindlimb unloading does not affect this cycle [26]; thus, the variation in sex-related hormones should have been averaged across the female group. In addition, we did not examine the muscle fiber type specificity, fiber size, and functional aspects of the other limb muscles owing to the relatively small rate of atrophy. Thus, we should consider long-term muscle adaptation of the hindlimb and the protective effect of losartan treatment on the different fiber types, muscle fiber size, and muscle function in future studies. Furthermore, the mechanisms underlying losartan-induced protection against TGF-β signaling activation were not fully investigated; hence, future work should incorporate several time points following hindlimb unloading (3, 5, or 14 days), which may help to fully elucidate the molecular mechanisms underlying losartan-induced protection against canonical and non-canonical TGF-β signaling. Finally, we could only show partial protection against muscle atrophy; therefore, future studies must also clarify the mechanisms underlying sex-specific protection against muscular atrophy using equivalent doses of losartan.

## Conclusions

Collectively, our results suggest that the blockade of the AT1 receptor with losartan has beneficial effects in disuse-induced skeletal muscle atrophy in female rats by preventing canonical TGF-β signaling, and provide insight into novel treatment strategies against muscle wasting in females. In contrast, losartan treatment did not alter the atrophic outcomes in male rats, which suggests sex-related differences in the response to AT1 receptor blockers during skeletal muscle atrophy. The sex-specificity for these observed effects could not be ascertained as different doses were used for males and females. Further investigations using the same dose for both sexes are warranted to elucidate the mechanism underlying the sex-specific differences observed in TGF-β signaling during hindlimb unloading.

### Supplementary Information

**Additional file 1: Figure S1.** Angiotensin II type I receptor protein expression at days 1 (A) and 7 (B) of hindlimb unloading, and representative blots.

**Additional file 2: Table S1.** Losartan administration per rat.

## Data Availability

All data generated or analyzed during this study are included in this published article [and its supplementary information files].

## Abbreviations

TGF-β: Transforming growth factor-beta
AT1: Angiotensin II type 1
CSA: Cross-sectional area
EDTA: Ethylenediaminetetraacetic acid
GDF: Growth differentiation factor
BSA: Bovine serum albumin
ANOVA: Analysis of variance
ActRIIB: Activin type IIB receptor
