# Supplementary material for: Losartan treatment attenuates hindlimb unloading-induced atrophy in the soleus muscle of female rats via canonical TGF-β signaling
Source: J Physiol Sci. 2022 Mar 9;72:6. doi: 10.1186/s12576-022-00830-8 (PMC10717208; doi:10.1186/s12576-022-00830-8)
Supplement: Supplementary file 1 — Additional file 1: Figure S1. Angiotensin II type I receptor protein expression at days 1 (A) and 7 (B) of hindlimb unloading, and representative blots. [file 12576_2022_830_MOESM1_ESM.doc]

**Figure S1. Angiotensin II type I receptor protein expression at days 1 (A) and 7 (B) of hindlimb unloading, and representative blots.** CON; control, LOS; losartan. Samples were collected before (PRE), and at days 1 and 7 after hindlimb unloading. Values are expressed as mean ± standard deviation; n = 7 per time point. For non-normal distribution, Kruskal-Wallis test was performed.
