# Supplementary material for: Losartan treatment attenuates hindlimb unloading-induced atrophy in the soleus muscle of female rats via canonical TGF-β signaling
Source: J Physiol Sci. 2022 Mar 9;72:6. doi: 10.1186/s12576-022-00830-8 (PMC10717208; doi:10.1186/s12576-022-00830-8)
Supplement: Supplementary file 2 — Additional file 2: Table S1. Losartan administration per rat. [file 12576_2022_830_MOESM2_ESM.doc]

**Table S1. Losartan administration per rat.**

| Day 1 | Total Losartan  Administration (g) | Losartan  Dosage (mg/kg/day) |  | Day7 | Total Losartan  administration (g) | Losartan  Dosage (mg/kg/day) |
| --- | --- | --- | --- | --- | --- | --- |
| Male rat #1 | 10.4 | 44.5 |  | Male rat #8 | 41.9 | 26.6 |
| Male rat #2 | 13.3 | 55.3 |  | Male rat #9 | 42.5 | 28.6 |
| Male rat #3 | 9.6 | 41.1 |  | Male rat #10 | 41.0 | 25.2 |
| Male rat #4 | 10.4 | 45.1 |  | Male rat #11 | 39.2 | 25.1 |
| Male rat #5 | 11.7 | 49.5 |  | Male rat #12 | 35.8 | 23.8 |
| Male rat #6 | 13.3 | 53.7 |  | Male rat #13 | 44.2 | 28.0 |
| Male rat #7 | 11.7 | 46.7 |  | Male rat #14 | 39.0 | 23.8 |
| ***Average (SD)*** | ***11.5 (1.4)*** | ***48.0 (5.1)*** |  | ***Average (SD)*** | ***40.5 (2.8)*** | ***25.9 (1.9)*** |
| Female rat #1 | 10.5 | 73.3 |  | Female rat #8 | 48.2 | 51.6 |
| Female rat #2 | 10.0 | 68.1 |  | Female rat #9 | 48.6 | 47.8 |
| Female rat #3 | 9.2 | 62.9 |  | Female rat #10 | 45.1 | 45.2 |
| Female rat #4 | 11.2 | 78.5 |  | Female rat #11 | 23.0 | 26.4 |
| Female rat #5 | 14.7 | 100.7 |  | Female rat #12 | 46.2 | 46.2 |
| Female rat #6 | 14.4 | 99.9 |  | Female rat #13 | 38.2 | 40.9 |
| Female rat #7 | 8.5 | 54.4 |  | Female rat #14 | 44.4 | 43.4 |
| ***Average (SD)*** | ***11.2 (2.4)*** | ***76.8 (17.8)*** |  | ***Average (SD)*** | ***42.0 (9.0)*** | ***43.1 (8.1)*** |

SD, standard deviation.
